# Two major gate-keepers in the self-renewal of neural stem cells: Erk1/2 and PLCγ1 in FGFR signaling

**DOI:** 10.1186/1756-6606-2-15

**Published:** 2009-06-08

**Authors:** Jin-A Lee, Deok-Jin Jang, Bong-Kiun Kaang

**Affiliations:** 1 Department of Biotechnology, College of Life Science and Nano Technology, Hannam University, 461-6, Jeonmin-dong, Yuseong-gu, Dajeon 305-811, Korea; 2 Department of Biological Sciences, National Creative Research Initiative Center for Memory, College of Natural Sciences, Seoul National University, 599 Gwanak-ro, Gwanak-gu, Seoul 151-747, Korea; 3 Department of Brain and Cognitive Sciences, National Creative Research Initiative Center for Memory, College of Natural Sciences, Seoul National University, 599 Gwanak-ro, Gwanak-gu, Seoul 151-747, Korea

## Abstract

Neural stem cells are undifferentiated precursor cells that proliferate, self-renew, and give rise to neuronal and glial lineages. Understanding the molecular mechanisms underlying their self-renewal is an important aspect in neural stem cell biology. The regulation mechanisms governing self-renewal of neural stem cells and the signaling pathways responsible for the proliferation and maintenance of adult stem cells remain largely unknown. In this issue of *Molecular Brain *[Ma DK et al. Molecular genetic analysis of FGFR1 signaling reveals distinct roles of MAPK and PLCγ1 activation for self-renewal of adult neural stem cells. Molecular Brain 2009, 2:16], characterized the different roles of MAPK and PLCγ1 in FGFR1 signaling in the self-renewal of neural stem cells. These novel findings provide insights into basic neural stem cell biology and clinical applications of potential stem-cell-based therapy.

## Self-renewal in neural stem cells

Stem cells have two fundamental properties: self-renewal and multipotency. Self-renewal is a cooperative process involving the proliferation and maintenance of an undifferentiated state. The loss of self-renewal properties leads to loss in multipotency, differentiation, and a change in cell fates. Therefore, self-renewal is tightly controlled by the dynamic interplay between intrinsic genetic factors (transcription factors, microRNA, and epigenetic control) and extrinsic factors from the microenvironments (niches) in which stem cells are generated, migrated, and located [1-4]. Among regulatory factors involved in self-renewal, FGF-2 – which primarily acts via the FGF receptor 1 (FGFR1) – is a well-known extrinsic factor maintaining the self-renewal ability of neural stem cells [5,6]. Neural stem cells are found in the adult brain as well as in developing brains, which suggests the essential roles of neurogenesis in adult brain functions [7]. Neurogenesis occurs in response to various environmental signals, such as injury, learning, and memory, or pathological stimuli, which contribute to the remodeling of brain tissues, homeostasis, and regeneration of tissues after an injury [8-10].

Many extrinsic factors such as EGF, VEGF, Wnt, Shh, receptor tyrosine kinases, and FGF-2 have been implicated in the self-renewal and differentiation of neural stem cells [11]. Adult neurogenesis depends greatly on FGF-2 signaling [12]. Although the studies on FGF-2 and its role in the maintenance or differentiation of neural stem cells are extensive, the mechanism by which FGFR signaling regulates self-renewal as well as the downstream signaling pathways contributing to the regulation of self-renewal in adult neural stem cells are not well elucidated thus far.

Ma et al. [13] have addressed these important issues by characterizing the differential roles of MAPK and PLCγ1 in FGFR1 signaling in the self-renewal of neural stem cells by using a chimeric TrkA-FGFR1.

## Generation of chimeric TrkA-FGFR1 receptor

First, to identify the cytoplasmic signaling pathways of FGFR1 by FGF-2 in the self-renewal of neural stem cells, the authors generated a chimeric receptor that combines the extracellular domain of NGF, whose receptor (TrkA) is hardly detectable in neural stem cells, with the cytoplasmic domain of FGFR1, which is one of the major receptors in neural stem cells [12,14]. To elucidate the downstream signaling of the FGFR1 involved in self-renewal by using TrkA-FGFR1, the authors' idea was that treatment of NGF instead of FGF-2 could mimic FGFR1 signaling and maintain the self-renewal process of neural stem cells. This novel idea allowed the authors to characterize and analyze the cytoplasmic signaling pathway of FGFR1 without affecting the endogenous self-renewal process by FGF-2 treatment.

In this study, the treatment of NGF recapitulated FGFR1 signaling via a recombinant TrkA-FGFR1 receptor in the self-renewal of stem cells. It is well-known that activation of FGFR can trigger multiple intracellular signaling cascades, including at least two independent pathways. One is associated with the Src-Homolgy 2 (SH2)-linked pathway mediated by PLCγ1, Crk, or Src, while the other pathway is linked to the FRS2-Ras signaling pathway [12,15,16].

What the authors found was that two amino-acid residues, L442 and Y766, which linked the downstream Ras-MAPK and PLCγ1, were essential for maintaining adult neural stem cells. While Ras-MAPK is necessary for growth-factor-induced, cell-cycle progression, PLCγ1 identified as the molecule associated with FGFR had not been well-demonstrated in proliferation and differentiation [17]. Therefore, the next important questions will be the potential role of Ras-MAPK or PLCγ1 in the regulation of the self-renewal of adult neural stem cells. To address this question, Ma et al. [13] identified the exact roles of each downstream signal pathway in the self-renewal of neural stem cells.

## Two distinct molecular players by FGFR1 signaling in the self-renewal of neural stem cells

### 1) ERK1/2 in FGF-2 signaling: proliferation and anti-neuronal differentiation

To define the role of MAPK downstream signaling following FGFR1 activation in the self-renewal of neural stem cells, Ma et al. [13] examined the effects of ERK1/2 on the proliferation and spontaneous differentiation of neural stem cells. In that study, the inhibition of ERK1/2 activation by U0126 or the expression of the dominant negative form of MEK1 reduced the proliferation and induced a spontaneous differentiation into the neuronal or glial cells. In contrast, the activation of MEK1 by the expression of the constitutive active form of MEK1 blocked spontaneous and induced neural differentiation by strongly supporting the assertion that ERK1/2 is necessary and sufficient for the proliferation of adult NSC. The authors detected the molecular pathway by MAPK-ERK signaling under FGFR1 activation by examining the expression of NeuroD1, an essential transcription factor for neuronal differentiation, or Cyclin D2, the key gene of cell cycle progression [18,19]. The authors found that the expression of NeuroD1 was reduced and that of Cyclin D2 was enhanced in the MEK1 CA expressing cell line by indicating that MAPK-ERK might regulate the proliferation and inhibition of spontaneous differentiation through the modulation of the gene expression of NeuroD1 or Cyclin D2.

In the future, it will be interesting to identify how MAPK-ERK signaling following FGFR activation is linked to the regulation of the expression of different sets of genes involved in proliferation and differentiation, especially considering how the specific modulation of the signaling pathway contributed to the self-renewal process for the clinical application of neural stem cell-based therapy. Moreover, Ma et al. [13] proposed notch signaling to be involved in the anti-differentiation effect of the MEK1 pathway. However, the exact cellular and molecular mechanisms linking the anti-differentiation effect and the notch pathway in MAPK signaling the under activation of FGFR1 remain to be investigated.

### 2) PLCγ1 in FGF-2 signaling: Maintaining the capacity for differentiation

The authors then examined the effect of PLCγ1, which is another potential regulatory signaling involved in the self-renewal of neural stem cells under FGFR activation.

They used shRNA technique and lipase-inactive dominant negative mutant of PLCγ1 for studying the loss of function in adult neural stem cells. In their study, the depletion of PLCγ1 decreased the number of GFAP^-^Nestin^+ ^cells – which are adult neuronal precursor cells – in normal proliferation conditions without affecting overall proliferation. Depletion of PLCγ1 led to a loss of identity of neural stem cells and resulted in the differentiation of neural stem cells into astrocytes. Ma et al. [13] found that PLCγ1 depleted cells mostly differentiated into GFAP^+ ^positive astrocytes, even in normal differentiation conditions, by impairing the capacities of neuronal and oligodendroglial differentiations. These novel findings highlight the role of PLCγ1 in the maintenance of self-renewal of neural stem cells and in the commitment of adult neural stem cells into astrocytes under undifferentiated or differentiated states. The study by Ma et al. [13] identified PLCγ1 signaling as a new molecular player in adult neural stem cells, which directs cell-fate decisions toward astrocyte formation.

Future studies on the mechanism by which PLCγ1 regulates cell-fate decisions toward astrocyte formation are warranted, in order to understand the mechanism underlying adult neurogenesis as well as glioblastoma which shows an impairment of neuronal and oligodendroglial differentiation [20].

## Conclusion and future perspectives

Multiple lines of evidence in the study by Ma et al. [13] show that the roles of major keepers in the proliferation and maintenance of adult neural stem cells are distinctly played by ERK1/2 and PLCγ1. ERK1/2 activation leads to proliferation and inhibits the spontaneous or induced differentiation of adult neural stem cells. Activated PLCγ1 maintains the full potential of adult neural stem cells to differentiate into neuronal or oligodendroglial cells. This outstanding work provides important insights with regard to cell therapy using neural stem cells and basic neural stem cell biology.

In brain regeneration therapies, the most important issue is to understand the mechanisms underlying the expansion and maintenance of stem cells. Recently, induced pluripotent stem cells and human embryonic stem cells were indicated as the main sources for this clinical application. For stem cell transplantation, appropriate protocols for differentiating pluripotent stem cells into neural stem cells and for aiding neural stem cell expansion *in vitro *should be considered in terms of the expansion of progenitor cells without over-proliferation, cell-fate determination, and integration of progenitor cells *in vivo*. Since FGF-2 and heparin are commonly used to maintain and expand neural progenitors, the insight on the role of FGFR in the downstream signaling pathway as provided in this novel study enables us to identify new targets whose modification is likely to improve protocols used to expand neural stem cells during transplantation therapy. These targets could also be manipulated in the brain to activate adult neurogenesis.

These findings, however, are based purely on an *in vitro *system using adult neural stem cells from the dentate gyrus of hippocampus. There is a debate of limited effect of FGF-2 in vivo in contrast with the ability of FGF-2 to maintain the self-renewal of neural stem cells *in vitro *[21]. Therefore, these novel pathways in FGFR signaling need to be investigated in adult neural stem cells *in vivo *under physiological or pathological microenvironments. Regardless of the need for further investigation in this study, the novel findings described by Ma et al. [13] in this issue of the *Molecular Brain *will certainly spur more in-depth studies underlying the self-renewal of adult neural stem cells. Furthermore, these future studies will likely move us closer to our goal of a clinical application for neural stem-cell-based therapy.
